# Predicting Antitumor Activity of Peptides by Consensus of Regression Models Trained on a Small Data Sample

**DOI:** 10.3390/ijms12128415

**Published:** 2011-11-29

**Authors:** Andreja Radman, Matija Gredičak, Ivica Kopriva, Ivanka Jerić

**Affiliations:** 1 Division of Organic Chemistry and Biochemistry, Ruđer Bošković Institute, Bijenička cesta 54, Zagreb HR-10000, Croatia; E-Mails: radman.andreja@gmail.com (A.R.); matija.gredicak@irb.hr (M.G.); 2 Division of Laser and Atomic Research and Development, Ruđer Bošković Institute, Bijenička cesta 54, Zagreb HR-10000, Croatia; E-Mail: ivica.kopriva@irb.hr

**Keywords:** opioid growth factor (OGF), QSAR descriptors, consensus of predictors

## Abstract

Predicting antitumor activity of compounds using regression models trained on a small number of compounds with measured biological activity is an ill-posed inverse problem. Yet, it occurs very often within the academic community. To counteract, up to some extent, overfitting problems caused by a small training data, we propose to use consensus of six regression models for prediction of biological activity of virtual library of compounds. The QSAR descriptors of 22 compounds related to the opioid growth factor (OGF, Tyr-Gly-Gly-Phe-Met) with known antitumor activity were used to train regression models: the feed-forward artificial neural network, the *k*-nearest neighbor, sparseness constrained linear regression, the linear and nonlinear (with polynomial and Gaussian kernel) support vector machine. Regression models were applied on a virtual library of 429 compounds that resulted in six lists with candidate compounds ranked by predicted antitumor activity. The highly ranked candidate compounds were synthesized, characterized and tested for an antiproliferative activity. Some of prepared peptides showed more pronounced activity compared with the native OGF; however, they were less active than highly ranked compounds selected previously by the radial basis function support vector machine (RBF SVM) regression model. The ill-posedness of the related inverse problem causes unstable behavior of trained regression models on test data. These results point to high complexity of prediction based on the regression models trained on a small data sample.

## 1. Introduction

Peptides are attracting increasing attention and have growing significance as therapeutics. They are Nature’s toolkit known to control and direct various cellular functions and intercellular communication events. For many years, peptide-based therapeutics were only considered for hormonal disorders and hormone-dependent cancers. However, novel technologies comprising synthetic procedures (solid-phase synthesis), recombinant processes and especially recent progress in drug delivery technologies, overcome many of the former drawbacks associated with peptide-based drugs [1,2]. About half of the peptides in clinical trials address oncology, metabolic, infectious and cardiovascular diseases-related targets. However, it is expected in the future that peptide drugs will address other medical disorders as well. Peptides offer several advantages over “classical” small molecules (higher specificity/selectivity, lower toxicity and tissue accumulation) or antibodies (smaller size, lack of serious immune responses, easy storage). Some of the most applied peptide-based drugs today are glatiramer acetate for the treatment of multiple sclerosis [3], leuprolide acetate, a GnRH receptor agonist for the treatment of breast and prostate cancers [4] and exenatide, approved for the treatment of diabetes mellitus type 2 [5].

Among short peptides with significant therapeutic potential, the native opioid growth factor (OGF), Met-enkephalin (Tyr-Gly-Gly-Phe-Met) is of particular interest. Numerous studies revealed that it acts in a receptor-mediated fashion and has regulatory function in the onset and progression of different human cancers [6]. OGF binds to the OGF receptor (OGFr) and modulates cyclin-dependent kinase inhibition pathway. Cell proliferation can be reduced by the increase of the OGF-OGFr activity through the addition of exogenous OGF [7] or some immunomodulators, like resiquimod, an upregulator of the OGFr [8]. Recent studies under the phase II clinical trials showed that biotherapy with OGF improves clinical benefits and even survival in patients with advanced pancreatic cancer [9], while the combination of chemotherapy with gemcitabine and biotherapy with OGF decreases pancreatic cancer growth and also reduced toxic effects of chemotherapy (*in vitro* experiments and animal models) [10].

The main drawback of OGF is low enzymatic stability and thus rapid hydrolysis in biological fluids. Some of the recent attempts to overcome this limitation involved incorporation of unnatural, adamantane-containing amino acids into primary OFG sequence [11]. It was found that the replacement of Gly^2^ with (*R,S*)-(1-adamantyl)glycine (Ada) gave the most effective derivative with antitumor activity against HEp-2, HBL, SW-620 and Caco-2 cell lines *in vitro*. Afterwards, the support vector machines (SVM) QSAR approach was undertaken to screen a virtual library of OGF-related compounds and identify novel structures with possibly improved antitumor activities [12]. Some of the top-rated compounds obtained by computational prediction were synthesized and showed more pronounced activity on the selected cancer cell lines. SVM approach is one of the most used QSAR models in rational drug design for the active/non-active classification problem. Additionally, probability based- and artificial neural networks (ANN) regression models were applied on similar problems [13–15]. The size of the training set determinates quality of prediction and in examples mentioned above it ranges from 100 to 1400 compounds. A common problem within the academic community is availability of a limited number of samples with measured biological activity. Thus, reliable identification of novel lead compound(s) from a virtual library becomes a challenging problem. The situation is generally known as the “small *N* large *p*” problem [16], and is very common in medicine, bioinformatics, computational drug design, etc. Therefore, methodology for the selection of regression model(s) that can possibly yield reliable and stable prediction is of crucial importance. Stability implies that predictions do not change significantly if small number of compounds, or even one, with measured activity is replaced in the training sample. Thus, it is the aim of the present work to validate potential of methodology that uses consensus between regression models in predicting biological activity of virtual compounds. Therefore, we have trained the following regression models: the feed forward ANN, the *k*-nearest neighbor (KNN), the sparseness constrained linear regression, the linear SVM and nonlinear SVM with polynomial and Gaussian kernels on a dataset of 22 OGF-related peptides with measured antitumor activity. Learned models were then applied on a virtual library consisted of 429 peptides yielding six lists with candidate compounds ranked by predicted antitumor activity. Consensus between the ranking lists has been sought. By using the majority voting principle a final ranking list has been obtained. The highly ranked candidate compounds were synthesized, characterized and tested for an antiproliferative activity. The activities were compared against compounds selected previously from the same virtual library by the radial basis function SVM regression model [12].

## 2. Results and Discussion

### 2.1. Regression Model Selection for Compound Activity Prediction

The training sample composed of 22 compounds with measured biological activity and 1,647 molecular descriptors (features) generated by the QSAR methodology to characterize each compound (details in the Experimental Section). Thus, the training data are described with the set: {**x**_n_ ∈ R^1647^, *y*_n_ ∈ R}_n=1_^22^ or in matrix formulation with **X** ∈ R^22 × 1647^ and **y** ∈ R^22^. When using stratified cross-validation (CV) to optimize prediction models small data sample with the large number of features yields CV error with large variance [17]. Since too few labeled data are available to distinguish role of different features in error analysis, selection of the parameters of regression models is uncertain [18–20].

In order to possibly counteract overfitting problems, we have trained six regression models listed previously. Prior to training and regression all compound descriptors were standardized to zero mean and unit variance whereupon the compound activity values **y** were scaled to [−1, 1] interval. Regression models were trained by leave-one-out and leave-two-out CVs using 1000 random partitions. The mean correlation between true and predicted values has been used as performance measure. The results of CV analysis are reported in Table S1 in the Supplementary Information. Sparse regression model was the only one with the mean correlation above 0.7 in both CV modes and the ANN model was closed to that. Linear SVM also has correlation value above 0.7 in leave-one-out CV. The RBF SVM had quite low correlation value, but top 5 compounds predicted by it agreed with the top 5 compounds predicted by models with better CV performance (see below). Among used regression models, only sparse linear regression model performs feature selection during the training process. That was expected to increase chance of predicting compound with high biological activity. This choice follows the principle of parsimony, also known as Occam’s razor, since the experimental data are explained with a less complex model (smaller number of features). Hence, overfitting should be reduced substantially.

Sparse regression model **w** is obtained as solution of the underdetermined system of linear equations **y** = **X w** + **e**, where **e** represents modeling error: $w=\underset{z}{\text{arg}\;\text{min}}\;\frac{1}{2}||y-\mathrm{Xz}|{|}_{2}^{2}+\;\lambda ||z|{|}_{1}$ and λ represents regularization constant. Its value depends on the noise variance. The least absolute shrinkage and selection operator (LASSO) [21] is such regression model. However, over the last decade a number of methods for sparse solution of the linear inverse problems with the formulations equivalent to above, have been developed [22–24] and conditions necessary for uniqueness of the solution are established. To be more specific, let us suppose that *k* = |**w**|_0_, *i.e.*, **w** has only *k* important (non-zero) coefficients that point out to corresponding molecular descriptors. Unique solution of **y** = **X w** in the problem considered here is obtained when: *k* < 22 and 22 ≈ *k* log 1647/*k*. Maximal number of molecular descriptors *k* that satisfies above inequality is 3. It is, however, not likely that compounds can be characterized with 3 QSAR molecular descriptors only. Some of the well conducted studies on the prediction of antibacterial activity of polypeptides were based on 44 [14] and 20 “inductive” QSAR descriptors [15]. Moreover, the prediction error ||**y**–**Xw||**_2_^2^ can’t be made reasonably small with such a small number of molecular descriptors. The interior point method [24] used to solve related inverse problem yielded regression vector with 7 coefficients that are significantly different than zero. But *k* = 7 implies that 39 samples (equations) with measured activity should be available to guarantee uniqueness and, consequently, stable behavior of learned regression model on test data. Evidently, that was impossible to achieve with such a small training dataset. The unstable behavior is demonstrated in disagreement between features that correspond with seven large coefficients of the regression vector (molecular descriptors GMTI, w, D/D, SRW07, SRW09, IDET and SHP2) and features (piID, MATS4p, Mor20m—see also Table S2 in the Supplementary Information) that are highly correlated (above 0.7) with the prediction of this sparse regression model on test data. This unstable behavior has been already recognized and discussed in gene selection for cancer prediction [25].

From the mathematical standpoint there is analogy between antitumor activity prediction in chemoinformatics and cancer prediction in bioinformatics. Thus, some advanced concepts used in microarray data analysis could potentially be beneficial in the problem considered here. Consensus between feature selection algorithms has been used very recently in microarray data analysis to alleviate disagreement between them and improve credibility of selected features (genes) [26]. Therefore, to counterattack uncertainty in selection of regression models, consensus between them has been sought after they were applied to unseen data.

Trained regression models were applied to 429 compounds from the virtual library (details regarding construction of the virtual library are given in the Experimental Section). For each regression model a list was formed with compounds ordered by predicted activity. Then we looked at lists that overlap significantly in top ten compounds. This has been achieved with the lists corresponding with the linear SVM, sparse regression model, feed forward ANN and RBF SVM (Table 1). Moreover, these four regression models yielded highly correlated predictions when applied to library of 429 virtual compounds with the correlation value above 0.94 (Table 2). Rankings of first 10 compounds in each of four lists were combined using the majority voting and yielded a final list where the first position has been reached with the minimal score. The final list of first 15 compounds is presented in Table 1 together with the top 10 compounds predicted by [12]; compound **10** had an overall score equal to 4 implying that it was first on the list of each of four regression models. The first 5 compounds were in the top 10 compounds in each of the four lists. It is important to point out that structures of these 5 compounds differ significantly from those predicted by the SVM Gaussian kernel [12]. This finding prompted us to synthesize compounds **10–14**, evaluate their biological potential and finally compare utilities of different regression models for a given problem.

### 2.2. Peptide Synthesis

**Figure 1:**
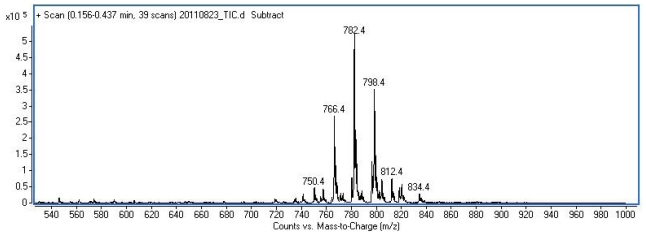
Part of the full scan mass spectrum of the reaction mixture during the deprotection of **8** showing presence of oxidized forms of methionine.

**Scheme 1:**
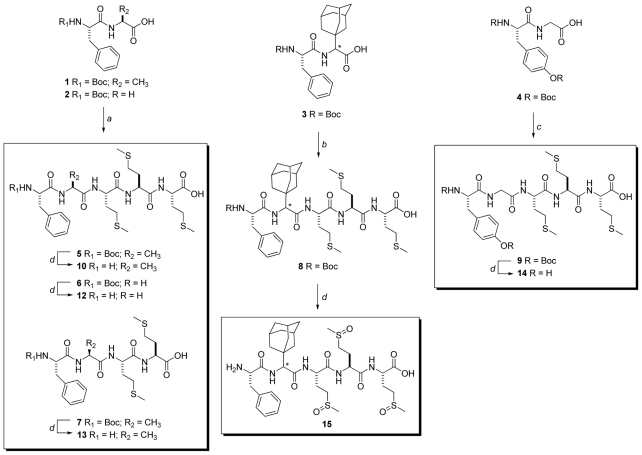
(**a**) HOSu (1.1 eqv), DCC (1.2 eqv), KHCO_3_ (1.2 eqv), then H-Met-Met-Met-OH (for **5** and **6**) or H-Met-Met-OH (for **7**) (1.1 eqv) in DMF; (**b**) NMM (1.2 eqv), HATU (1.2 eqv), then H-Met-Met-Met-OH (1.2 eqv) in DMF; (**c**) NMM (2.3 eqv), ClOCOiBu (1.2 eqv), H-Met-Met-Met-OH (1.1 eqv) in DMF; (**d**) TFA-H_2_O (9:1).

Synthesis of methionine rich peptides **10–14** was based on the 2 + 2 and 2 + 3 coupling between Boc-protected dipeptides **1–4** and H-Met-Met-OH or H-Met-Met-Met-OH (Scheme 1). Different methods of peptide bond formation were tested; finally peptides **5–7** were obtained by the DCC/HOSu method, activation with HATU yielded Ada-containing pentapeptide **8**, while pentapeptide **9** was obtained by the mixed anhydride method (details in the Experimental section). Finally, deprotection of the Boc group was performed by acid hydrolysis and extra precautions were taken due to the known susceptibility of methionine residue toward acid-promoted oxidation to sulfoxide. Therefore, deprotection step was performed in ice bath, crude product was immediately purified by the HPLC and residual acid removed by passing through the short C18 column. This procedure hampered oxidation of Met residue in all derivatives with the exception of Ada containing pentapeptide **11**, which showed extreme susceptibility to oxidation. The full scan mass spectrum of product(s) obtained in deprotection step (Figure 1) clearly revealed presence of only traces of **11** (*m/z* 750) and all three possible oxidized forms of **11** (*m/z* 766, 782 and 798). During the purification procedure, only oxidized product **15** was isolated. All prepared compounds were characterized by NMR spectroscopy and deprotected compounds **10** and **12–15** additionally by tandem mass spectrometry and HRMS. The main feature of MS/MS spectra of the molecular [M + H]^+^ ions of all peptides is high abundance of Y” and B-type fragment ions (Figure S1, Supplementary Information).

### 2.3. Proliferation Assay

Peptides **10**, **12–14** as well as trisulfoxide derivative **15** were screened for possible antiproliferative effects on three cell lines: SW 620 (colon carcinoma), MCF-7 (breast carcinoma) and HeLa (cervical carcinoma), according to the previously published procedure [12]. Table 3 summarizes activities found for tested peptides, expressed as percentage of growth (PG) of three cell lines, together with values for OGF and three OGF-related peptides proposed by the SVM radial basis function kernel (RBF) [12]. *In vitro* screening results confirmed that some of tested compounds possess more pronounced activity compared to the OGF on certain cell lines (numbers in bold, Table 3). However, compared to three OGF-related peptides proposed by the SVM radial basis function kernel (RBF), compounds predicted by the consensus of regression models proposed herein are less effective in inhibition of tumor cells growth. Comparison with the most active peptides from the training set revealed comparable antiproliferative effects on the SW620 cell line, but improved activities on HeLa and especially MCF-7 cell lines (Table 3). Therefore, the approach presented here managed to select peptides with improved biological activities from virtual library.

Regarding amino acid sequence, peptides proposed by the consensus of regression models differ significantly from those prepared following the RBF SVM regression model prediction [12]. Only one derivative in top-five contains non-natural adamantane-related amino acid, and all are rich in methionine. Contrary to that, prevalence of phenylalanine is characteristic of peptides described in [12]. Improved biological activity was attributed to the hydrophobic character and somewhat fixed conformation concluded from CD spectra. Presence of Ada, a γ-turn inducer, contributes to the observed conformational change when compared with the native OGF. Peptides prepared according to the consensus of regression models are less hydrophobic (log *P* values, Table 1), and absence of bulky Ada residue probably influences flexibility of prepared peptides. On the other hand, in peptides prepared in [12] third position was occupied with Gly, which allow flexibility of the peptide chain and thus help accommodation of the rigid Ada amino acid. It can be expected that, owing to the prevalence of nonpolar aliphatic amino acid methionine, peptides prepared in this work are structurally distinct from those prepared in [12] where aromatic amino acid phenylalanine is incorporated. Therefore, all these diversities can influence observed lower antiproliferative potential of tested peptides. Interestingly, trisulfoxide derivative **15** obtained by the oxidation of unstable, the only Ada-containing peptide **11**, showed similar antiproliferation potential as other peptides tested in this work, despite its unfavourable characteristics (log *P* −1.49, log *S* −3.03). Therefore, different structural and conformational factors contribute to biological profiles of studied peptides.

Although top 5 ranked compounds were on the lists of four regression models, compounds synthesized from that list were less effective in inhibition of tumor cell growth than RBF SVM predicted compounds in [12]. This confirms complexity and unreliability of prediction when regression models are trained on a sample with small number of compounds with measured antitumor activity and large number of features. This instability of prediction is consequence of the high ill-posedness of the concrete problem and is inherent to all “small *N* large *p* problems”, as shown in [25] for the gene subset selection problem. Thus, it is possibly wise to propose development of mathematical model for evaluation of the quality of prediction achieved by various statistical and machine learning methods in the spirit of the methods developed in [27] for validation of gene selection algorithms. It is also important to focus efforts and attention strictly on feature selection methods and try to reduce redundant features before training of regression models. Since reduction of features increases the ration between number of labeled samples and number of features this should up to some extent counteract instability of predictions on test data caused by overfitting. One such concept that combines L_1_ and L_2_ regularization has been proposed recently in [28].

## 3. Experimental Section

### 3.1. Construction of the Virtual Library

Details about training set, QSAR descriptors and virtual library construction are available at [29]. Web-site also contains structures of peptides in the virtual library, their molecular descriptors, and the predicted cytostatic activities. Briefly, 22 OGF-related peptides with measured antiproliferative activity were submitted to the E-Dragon web service [30], that predicts a probable three-dimensional structure using CORINA [31] and computes molecular descriptors for each compound. A virtula library was constructed encompassing OGF-like tri-, tetra- and pentapeptides. Amino acids present in training set peptides were used in the virtual library with two additional members to raise variety. At the N-terminal position, aromatic amino acids Tyr, Phe and Trp were used, at the second position Gly, Ala and Ada were varied, while other positions were occupied with Gly, Ala, Ada, Phe and Met. We used Marvin 5.1.1 software [32] to visualize, browse, and otherwise handle the molecules in the virtual library.

### 3.2. Software Environment

The regression models were implemented in MATLAB script language using functions available in the classification toolbox: newff, train and sim for the feed forward ANN, knnclassify for the KNN predictor and our own MATLAB implementation of the linear and nonlinear SVM-based regression models (files in MATLAB script language are given in Supplementary Information). The feed-forward ANN was composed of one input layer with ten nonlinear neurons (sigmoids) and one output layer with pure linear neuron. The network was trained in the backpropagation mode and the smallest CV-based prediction error has been obtained in the trainoss mode (the one step secant algorithm). Sparse linear regression was implemented by the interior point method [24], designed for the solution of large scale linear inverse problems with a MATLAB code available at [33].

### 3.3. Peptide Synthesis

#### 3.3.1. Materials and Methods

Reaction courses were monitored by TLC on Silica Gel 60 F_254_ Merck plates and examined under UV light or detected with HBr/ninhydrine. Column chromatography was performed with Merck silica gel (0.040–0.063 mm). Optical activities were measured on Optical Activity LTD automatic AA-10 polarimeter at 20 °C. Analysis was performed on HPLC system coupled with UV detector; C-18 semipreparative (250 × 8 mm, ID 5μm) column at flow rate 1 mL/min, or analytical (150 × 4.5 mm, ID 5 μm) column at flow rate 0.5 mL/min was used under isocratic conditions using different concentration of MeOH in 0.1% aqueous TFA. UV detection was performed at 254 or 280 nm. NMR spectra were recorded on 600 MHz and 300 MHz spectrometers, operating at 150.92 or 75.47 MHz for ^13^C and 600.13 or 300.13 MHz for ^1^H nuclei. TMS was used as an internal standard. Mass spectrometry measurements were performed on HPLC system coupled with triple quadrupole mass spectrometer, operating in positive electrospray ionization (ESI) mode. Spectra were recorded from a 10 μg/mL compound solution in 50% methanol/0.1% FA by injection of 2 μL into the ion source of the instrument by autosampler, at the flow rate of 0.2 mL/min (mobile phase 50% methanol/0.1% FA). HRMS analysis was performed on MALDI-TOF mass spectrometer operating in reflectron mode. Calibration type was internal with calibrants produced by matrix ionization (monomeric, dimeric and trimeric CHCA), azithromycin and angiotensin II dissolved in α-cyano-4-hydroxycinnamic acid matrix in the mass range *m/z* 190.0499 to 749.5157 or 1046.5417. Accurately measured spectra were internally calibrated and elemental analysis was performed on Data Explorer v. 4.9 Software with mass accuracy better than 5 ppm. Samples were prepared by mixing 1 μL of analyte methanol solution with 5 μL of saturated (10 mg/mL) solution of α-cyano-4-hydroxycinnamic acid (α-CHCA) and internal calibrants (0.1 mg/mL) dissolved in 50% acetonitrile/0.1% TFA. Synthesis of (*R,S*)-(1-adamantyl)glycine (Ada) and Boc-Phe-Ada-OH (4) is described previously [11].

#### 3.3.2. General Procedure for the Synthesis of Compounds **5**–**7**

Selected Boc-protected dipeptide **1** or **2** (Boc-Phe-Ala-OH or Boc-Phe-Gly-OH, respectively) (0.150 mmol) was dissolved in dry DMF at 0 °C. HOSu (0.180 mmol) and DCC (0.195 mmol) were added into the solution and the resulting mixture was stirred for 30 min at 0 °C and then at room temperature overnight. The reaction mixture was filtered and the precipitate washed with DMF. Mother liquor was added dropwise into the suspension of KHCO_3_ (0.198 mmol) and H-Met-Met-Met-OH or H-Met-Met-OH (0.180 mmol) in water. The reaction mixture was stirred for 3 hours at room temperature. pH of the solution was set to 2–3 with 10% citric acid and the product was extracted with Et_2_O, washed with brine and water and purified by flash chromatography.

#### 3.3.3. Boc-Phe-Ala-Met-Met-Met-OH (**5**)

Yield: 19% (colorless oil). R_f_ 0.22 (petrol ether-EtOAc-AcOH 10:10:0.5). [α]_D_ −10° (*c* 1, MeOH). ESI-MS: [M + H]^+^ *m/z* 730. ^13^C NMR (DMSO-*d*_6_): δ = 14.5 (SCH_3_ Met^3,4,5^), 18.4 (β Ala), 28.1 (CH_3_ Boc), 29.5, 29.6, 29.7 (β Met^3,4,5^), 31.8, 32.1, 33.3 (γ Met^3,4,5^), 37.3 (β Phe), 44.4 (α Ala), 51.6 (α Phe), 52.1, 53.0, 55.6 (α Met^3,4,5^), 78.5 (C Boc), 126.1 (ζ Phe), 127.9 (ɛ Phe), 129.2 (δ Phe), 138.1 (γ Phe), 155.3 (CO Boc), 169.9, 170.8, 172.3 (CO Met^3,4,5^), 171.3 (CO Phe), 171.4 (CO Ala). ^1^H NMR (DMSO-*d*_6_): δ =1.10–1.31 (m, 12H, CH_3_ Boc, β Ala), 1.92–2.10 (m, 15H, β Met^3,4,5^, SCH_3_ Met^3,4,5^), 2.21–2.52 (m, 6H, γ Met^3,4,5^), 2.72–2.92 (m, 2H, β Phe), 4.01–4.71 (m, 5H, α Phe, α Ala, α Met^3,4,5^), 6.94 (br d, 1H, NH Phe), 7.20–7.35 (m, 5H, ɛ, δ, ζ Phe), 7.72–7.85 (m, 1H, NH Met^3^), 7.91–8.34 (m, 3H, NH Ala, NH Met^4,5^).

#### 3.3.4. Boc-Phe-Gly-Met-Met-Met-OH (**6**)

Yield: 45% (colorless oil). R_f_ 0.18 (petrol ether-EtOAc-AcOH 10:10:0.5). [α]_D_ −17 ° (*c* 1, MeOH). ESI-MS: [M + H]^+^ *m/z* 716. ^13^C NMR (DMSO-*d*_6_): δ = 14.6 (SCH_3_ Met^3,4,5^), 24.4, 25.3 (β Met^3,4,5^), 28.1 (CH_3_ Boc), 29.4, 29.5, 29.6 (γ Met^3,4,5^), 37.3 (β Phe), 42.1 (α Gly), 47.5, 51.8 (α Met^3,4,5^), 55.7 (α Phe), 78.1 (C Boc), 126.1 (ζ Phe^1^), 127.9 (ɛ Phe), 129.2 (δ Phe), 138.2 (γ Phe), 155.2 (CO Boc), 168.7 (CO Phe), 170.5 (CO Met^3,4,5^), 172.8 (CO Gly). ^1^H NMR (DMSO-*d*_6_): δ =1.25–1.30 (m, 9H, CH_3_ Boc), 1.49–1.80 (m, 6H, β Met^3,4,5^), 1.94–2.04 (m, 9H, SCH_3_ Met^3,4,5^), 2.40–2.44 (m, 6H, γ Met^3,4,5^), 2.70–2.75, 3.00–3.01 (dd, 2H, β Phe), 3.70–3.74 (m, 2H, α Gly), 4.00–4.15 (m, 2H, α Phe, α Met^3^), 4.30–4.39 (m, 2H, α Met^4,5^), 6.96 (d, 1H, NH Phe), 7.18–7.26 (m, 5H, ɛ, δ, ζ Phe), 7.89, 7.97, 8.14 (br d, 3H, NH Met^3,4,5^), 8.20–8.22 (m, 1H, NH Gly).

#### 3.3.5. Boc-Phe-Ala-Met-Met-OH (**7**)

Yield: 54% (colorless oil). R_f_ 0.28 (petrol ether-EtOAc-AcOH 10:10:0.5). [α]_D_ −15 ° (*c* 1, MeOH). ESI-MS: [M + H]^+^ *m/z* 599. ^13^C NMR (DMSO-*d*_6_): δ = 15.0, 15.1 (SCH_3_ Met^3,4^), 18.6 (β Ala), 28.7 (CH_3_ Boc), 29.8, 30.0 (β Met^3,4^), 30.0, 31.3 (γ Met^3,4^), 37.4 (β Phe), 48.7 (α Phe), 51.9, 52.3 (α Met^3,4^), 56.2 (α Ala), 78.5 (C Boc), 126.6 (ζ Phe), 128.5 (ɛ Phe), 129.6 (δ Phe), 138.7 (γ Phe), 155.7 (CO Boc), 171.3 (CO Phe), 171.9, 172.5 (CO Met^3,4^), 173.6 (CO Ala). ^1^H NMR (DMSO-*d*_6_): δ =1.23–1.29 (m, 12H, CH_3_ Boc, β Ala), 1.80–2.00 (m, 10H, β Met^3,4^, SCH_3_ Met^3,4^), 2.41–2.62 (m, 4H, γ Met^3,4^), 2.71–2.96 (m, 2H, β Phe), 4.15–4.36 (m, 4H, α Phe, α Ala, α Met^3,4^), 6.94 (d, 1H, NH Phe), 7.19–7.26 (m, 5H, δ, ɛ, ζ Phe), 8.07 (m, 3H, NH Ala, NH Met^3,4^).

#### 3.3.6. Synthesis of Boc-Phe-Ada-Met-Met-Met-OH (**8**)

Boc-Phe-Ada-OH (**3**) (50 mg, 0.110 mmol), NMM (24 μL, 0.132 mmol) and HATU (50 mg, 0.132 mmol) were dissolved in DMF and stirred at room temperature for 2 hours. H-Met-Met-Met-OH (55 mg, 0.132 mmol) was added and the reaction mixture was stirred overnight. The solvent was evaporated in vacuo and the product was purified by flash chromatography in EtOAc-petrol ether-AcOH 10:5:0.5.

Yield: 63% (colorless oil). R_f_ 0.51 (EtOAc-petrol ether-AcOH 10:5:0.5). [α]_D_ −13° (*c* 1, MeOH). ESI-MS: [M + H]^+^ *m/z* 851. ^1^H NMR (CD_3_OD): δ = 1.42 (s, 9H, Boc), 1.56–1.80 (m, 12H, CH_2_Ada), 1.90–2.15 (m, 18H, CH Ada, β Met^3,4,5^, SCH_3_ Met^3,4,5^), 2.48–2.65 (m, 6H, γ Met^3,4,5^), 2.86–2.94; 3.10–3.19 (m, 2H, β Phe), 4.35–4.60 (m, 5H, α Phe, α Ada, α Met^3,4,5^), 7.17–7.35 (m, 5H, δ, ɛ, ζ Phe).

#### 3.3.7. Synthesis of Boc-Tyr(Boc)-Ala-Met-Met-Met-OH (**9**)

Boc-Tyr(Boc)-Ala-OH (**4**) (50 mg, 0.110 mmol) and NMM (28 μL, 0.254 mmol) were dissolved in DMF at 0 °C and ClOCOiBu (19 μL, 0.133 mmol) was added. After 10 min, H-Met-Met-Met-OH (50 mg, 0.122 mmol) was added and the reaction mixture was stirred for 30 min at 0 °C and then at room temperature overnight. The solvent was evaporated; water was added to the residue and pH adjusted to 2–3 with 10% citric acid. The product was extracted with EtOAc, washed with brine and water and purified by flash chromatography in petrol ether-EtOAc-AcOH 10:10:0.5.

Yield: 19% (colorless oil). R_f_ 0.19 (petrol ether-EtOAc-AcOH 10:10:0.5). [α]_D_ −7° (*c* 1, MeOH). ESI-MS: [M + H]^+^ *m/z* 847. ^13^C NMR (DMSO-*d*_6_): δ = 15.1 (SCH_3_ Met^3,4,5^), 18.6 (β Ala), 28.6, 28.7 (CH_3_ NHBoc, CH_3_ OBoc), 29.9, 30.2, 30.1 (β Met^3,4,5^), 32.1, 32.3, 32.9 (γ Met^3,4,5^), 37.1 (β Tyr), 48.5 (α Tyr), 49.1, 52.5, 54.0 (α Met^3,4,5^), 56.0 (α Ala), 78.5 (C OBoc), 83.5 (C NHBoc), 115.3 (ζ Tyr), 121.3 (ɛ Tyr), 130.6 (δ Tyr), 136.3 (γ Tyr), 149.6 (CO NHBoc), 151.8 (CO OBoc), 155.8, 170.1, 171.3 (CO Met^3,4,5^), 171.8 (CO Tyr), 171.4 (CO Ala). ^1^H NMR (DMSO-*d*_6_): δ =1.13–1.48 (m, 21H, CH_3_ OBoc, CH_3_ NHBoc, β Ala), 1.95–2.06 (m, 15H, β Met^3,4,5^, SCH_3_ Met^3,4,5^), 2.32–2.50 (m, 6H, γ Met^3,4,5^), 2.70–3.03 (m, 2H, β Tyr), 3.20–4.40 (m, 5H, α Tyr, α Ala, α Met^3,4,5^), 6.60 (d, 1H, NH Tyr), 6.80 (br d, 2H, ɛ Tyr), 7.10 (br d, 2H, δ Tyr), 7.20–8.22 (m, 4H, NH Ala, NH Met^3,4,5^).

#### 3.3.8. General procedure for the synthesis of compounds **10**–**14**

Boc-protected peptides **5–9** were treated with cold TFA-H_2_O (9:1) mixture in ice bath for 5 min. After addition of cold diethyl ether, the precipitate was collected by centrifugation, purified by HPLC and the residual TFA removed by passing through the short C18 column.

#### 3.3.9. H-Phe-Ala-Met-Met-Met-OH (**10**)

Yield: 25% (colorless oil). R_t_ 11.6 min (40% MeOH in 0.1% TFA). ESI-MS: [M + H]^+^ *m/z* 630. ^1^H NMR (CD_3_OD): δ = 1.46 (d, 3H, β Ala), 1.92–2.23 (m, 15H, β Met^3,4,5^, SCH_3_ Met^3,4,5^), 2.46–2.68 (m, 6H, γ Met^3,4,5^), 2.81–2.88; 2.95–3.01 (m, 2H, β Phe), 3.59, 3.69 (br d, 2H, α Me^t4,5^), 4.20–4.53 (m, 3H, α Phe, α Ala, α Met^3^), 7.20–7.36 (m, 5H, ɛ, δ, ζ Phe). HRMS (MALDI): *m/z* [M + H]^+^ calcd for C_27_H_43_N_5_O_6_S_3_ 630.2448, found 630.2456.

#### 3.3.10. H-Phe-Gly-Met-Met-Met-OH (**12**)

Yield: 20% (colorless oil). R_t_ 14.5 min (40% MeOH in 0.1% TFA). ESI-MS: [M + H]^+^ *m/z* 616. ^1^H NMR (CD_3_OD): δ = 1.93–2.00; 2.10–2.17 (m, 6H, β Met^3,4,5^), 2.06; 2.90; 2.10 (s, 9H, SCH_3_ Met^3,4,5^), 2.45–2.60 (m, 6H, γ Met^3,4,5^), 2.86–2.91, 3.11–3.15 (m, 2H, β Phe), 3.57 (br s, 1H, α Met^3^), 3.79; 3.92 (br d, 2H, α Gly), 4.24–4.28 (m, 1H, α Phe), 4.44–4.50 (m, 2H, α Met^4,5^), 7.21–7.33 (m, 5H, ɛ, δ, ζ Phe). HRMS (MALDI): *m/z* [M + H]^+^ calcd for C_26_H_41_N_5_O_6_S_3_ 616.22928, found 616.2286.

#### 3.3.11. H-Phe-Ala-Met-Met-OH (**13**)

Yield: 34% (colorless oil). R_t_ 17.1 min (40% MeOH in 0.1% TFA). ESI-MS: [M + H]^+^ *m/z* 499. ^1^H NMR (CD_3_OD): δ = 1.41 (d, 3H, β Ala, ^3^*J*_αβ_ = 7.1 Hz), 1.92–2.19 (m, 10H, β Met^3,4^, SCH_3_ Met^3,4^), 2.48–2.62 (m, 4H, γ Met^3,4^), 2.63–2.70 (m, 2H, β Phe), 4.04–4.08, 4.36–4.49 (m, 4H, α Phe, α Ala, α Met^3,4^), 7.28–7.40 (m, 5H, ɛ, δ, ζ Phe). HRMS (MALDI): *m/z* [M + H]^+^ calcd for C_22_H_34_N_4_O_5_S_2_ 499.2043, found 499.2028.

#### 3.3.12. H-Tyr-Ala-Met-Met-Met-OH (**14**)

Yield: 24% (colorless oil). R_t_ 20.9 min (43.5% MeOH in 0.1% TFA). ESI-MS: [M + Na]^+^ *m/z* 668. ^1^H NMR (CD_3_OD): δ = 1.41 (d, 3H, β Ala, ^3^*J*_αβ_ = 7.1 Hz), 1.85–2.10 (m, 15H, β Met^3,4,5^, SCH_3_ Met^3,4,5^), 2.50–2.63 (m, 6H, γ Met^3,4,5^), 2.85–3.10 (m, 2H, β Tyr), 4.04–4.08 (m, 1H, α Tyr) 4.36–4.49 (m, 3H, α Met^3,4^), 4.55–4.62 (m, 1H, α Ala), 6.80 (br d, 2H, ɛ Tyr), 7.10 (br d, 2H, δ Tyr). HRMS (MALDI): *m/z* [M + Na]^+^ calcd for C_27_H_43_NaN_5_O_7_S_3_ 668.2216, found 668.2211.

#### 3.3.13. H-Phe-Ada-Met(O)-Met(O)-Met(O)-OH (**15**)

Yield: 30% (colorless oil). R_t_ 15.9 min. (50.5% MeOH in 0.1% TFA). ESI-MS: [M + H]^+^ *m/z* 798. ^1^H NMR (CD_3_OD): *δ* = 1.50–1.82 (m, 12H, CH_2_Ada), 1.95–2.15 (m, 9H, CH Ada, β Met^3,4,5^), 2.45–2.65 (m, 15H, γ Met^3,4,5^, SOCH_3_ Met^3,4,5^), 2.80–2.95; 3.10–3.15 (m, 2H, β Phe), 4.10–4.16 (m, 1H, α Phe) 4.25–4.57 (m, 4H, α Ada, α Met^3,4,5^), 7.15–7.30 (m, 5H, δ, ɛ, ζ Phe). HRMS (MALDI): *m/z* [M + H]^+^ calcd for C_36_H_55_N_5_O_9_S_3_ 798.3233, found 798.3232.

### 3.4. Proliferation Assay

The biological potential of prepared compounds has been tested in the Laboratory for experimental therapy (Ruđer Bošković Institute) following the previously published procedure [12]. Briefly, MCF-7, SW 620 and HeLa cells were cultured as monolayers and maintained in Dulbecco’s modified Eagle medium (DMEM), supplemented with 10% fetal bovine serum (FBS), 2mM l-glutamine, 100 U/mL penicillin and 100 μg/mL streptomycin in a humidified atmosphere with 5% CO_2_ at 37 °C. The panel cell lines were inoculated onto a series of standard 96-well microtiter plates on day 0, at 1 × 10^4^ to 3 × 10^4^ cells/mL, depending on the doubling times of specific cell line. Test agents were then added in five, 10-fold dilutions (10^−8^ to 10^−4^ M) and incubated for a further 72 hours. Working dilutions were freshly prepared on the day of testing. The solvent was also tested for eventual inhibitory activity by adjusting its concentration to be the same as in working concentrations. After 72 hours of incubation the cell growth rate was evaluated by performing the MTT assay. For this purpose the substance treated medium was discarded and MTT was added to each well at a concentration of 20 μg/40 μL. After four hours of incubation the precipitates were dissolved in 160 μL of dimethylsulphoxide (DMSO). The absorbance that is directly proportional to the number of living, metabolically active cells was measured on a microplate reader at 570 nm. Each test point was performed in quadruplicate in three individual experiments.

## 4. Conclusions

The results of the present work demonstrate the complexity of prediction of biological activity of candidate molecules based on the regression models trained on a dataset of very small ratio between the number of labeled samples and number of features. Six regression models were trained on a dataset of 22 OGF-related peptides with measured antitumor activity (1647 QSAR descriptors) and then applied on a 429 virtual library members. To, possibly, hinder overfitting and instability of prediction, consensus among the regression models has been sought. Highly ranked compounds were prepared and tested. Although some of them showed more pronounced activity compared with the native OGF, they were less active than highly ranked compounds selected previously from the same virtual library by the RBF SVM regression model. The RBF SVM regression model has also been considered here and showed smaller cross-validation accuracy. However, its prediction has been a part of the consensus reached, in addition to RBF SVM, between feed-forward ANN, linear SVM and linear sparse regression model. This seemingly paradoxical result confirms inherent instability of prediction that is based on regression model trained on a (very) small sample size. This has been additionally confirmed by high correlation (0.94) of predictions of test data between these four models, whereas list of features that are highly correlated with predictions of corresponding models do not overlap. As this situation is, unfortunately, often found within the academic community, it is possibly wise to consider development of a mathematical model for the evaluation of the quality of prediction achieved under such conditions, in the spirit of the methods developed recently in microarray data analysis. It is also recommended to consider application of existing or development of novel feature selection methods to remove redundant features. This would increase the ratio between the number of labeled samples and number of features before training of regression models and, thus, should reduce instabilities of predictions of test data.

## Supplementary Information



## Figures and Tables

**Table 1:** Ranking list of the activity of virtual compounds as predicted by four regression models (linear SVM, sparse regression, MLP ANN, RBF SVM). Numbers in parentheses represent position of given compound on individual lists obtained by four regression models. Top-rated compounds predicted in reference [12] are given for comparison.

Rank/(position on the lists/mean value)	Compounds	MW	log *P*	log *S*
1. (1-1-1-1/1)	H-Phe-Ala-Met-Met-Met-OH (**10**)	629.96	0.89	−5.23
2. (2-3-2-4/2.75)	H-Phe-Ada-Met-Met-Met-OH (**11**)	750.17	1.12	−6.06
3. (5-2-4-3/3.25)	H-Phe-Gly-Met-Met-Met-OH (**12**)	615.93	0.76	−5.22
3. (4-4-3-2/3.25)	H-Phe-Ala-Met-Met-OH (**13**)	498.74	0.62	−4.70
5. (8-5-5-5/5.25)	H-Tyr-Ala-Met-Met-Met-OH (**14**)	645.96	0.58	−4.99
**Compounds below are listed on three lists only**				
6. (3-6-x -6/ 5)	H-Phe-Ada-Met-Met-OH	618.95	0.91	−5.70
7. (6-7-6 -x/ 6.33)	H-Tyr-Ada-Met-Met-Met-OH	766.17	0.72	−5.66
**Compounds below are listed on two lists only**				
8. (x-x-8-7/7.5)	H-Phe-Ala-Phe-Met-Met-OH	645.93	0.78	−5.42
9. (7-x-9-x/8)	H-Phe-Ada-Phe-Met-Met-OH	766.14	1.40	−5.77
10. (9-8-x-x/8.5)	H-Tyr-Ada-Met-Met-OH	634.95	0.62	−5.27
11. (x-x-7-10/8.5)	H-Phe-Ala-Met-Met-Gly-OH	555.80	0.80	−4.58
12. (x-10-x-8/9)	H-Phe-Gly-Met-Met-OH	484.71	0.58	−4.69
**Compounds below are listed on one list only**				
13. (x-9-x-x)	H-Tyr-Gly-Met-Met-Met-OH	631.93	0.50	−4.95
13. (10-x-x-x-x)	H-Phe-Ada-Met-Met-Gly-OH	676.01	0.83	−5.53
13.(x-x-x-9)	H-Tyr-Ala-Met-Met-OH	514.74	0.51	−4.26
13. (x-x-10-x)	H-Tyr-Ala-Met-Met-OH	514.74	0.51	−4.26
**Top-rated compounds predicted in [12]**				
H-Tyr-Ada-Gly-Phe-Met-OH	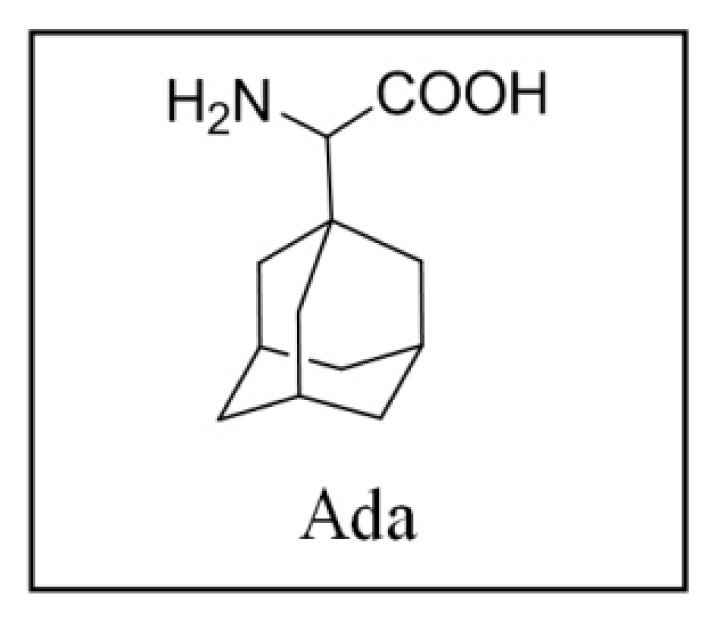	-	-	-
H-Phe-Ada-Gly-Phe-Met-OH				
H-Phe-Ada-Gly-Phe-Phe-OH				
H-Tyr-Ada-Gly-Phe-Phe-OH				
H-Trp-Ada-Gly-Phe-Met-OH				
H-Tyr-Ada-Gly-Phe-Gly-OH				
H-Trp-Ada-Gly-Phe-Phe-OH				
H-Phe-Ada-Gly-OH				
H-Phe-Gly-Gly-Phe-Phe-OH				
H-Phe-Gly-Aaa-Gly-OH				

**Table 2:** Correlation of predictions between regression models.

	sparse	Lin SVM	MLP	RBF SVM	Poly SVM	KNN
sparse	1.0	0.9701	0.9391	0.9406	0.5510	0.3275
Lin SVM	-	1.0	0.9631	0.9568	0.4860	0.3141
MLP	-	-	1.0	0.9359	0.4152	0.2685
RBF SVM	-	-	-	1.0	0.6278	0.3631
Poly SVM	-	-	-	-	1.0	0.1396
KNN	-	-	-	-	-	1.0

**Table 3:** Growth inhibition effects of OGF-related peptides.

Compound	PG(%) a		
	SW620	MCF-7	HeLa
H-Phe-Ala-Met-Met-Met-OH (**10**)	100	**74**	88
H-Phe-Gly-Met-Met-Met-OH (**12**)	95	86	86
H-Phe-Ala-Met-Met-OH (**13**)	86	**68**	**71**
H-Tyr-Ala-Met-Met-Met-OH (**14**)	97	**71**	91
H-Phe-Ada-Met(O)-Met(O)-Met(O)-OH (**15**)	77	82	**64**

H-Tyr-Gly-Gly-Phe-Met-OH (OGF)	**85**	**92**	**88**

H-Phe-Ada-Gly-Phe-Met-OH b	35	39	49
H-Phe-Ada-Gly-Phe-Phe-OH b	39	41	23
H-Tyr-Ada-Gly-Phe-Phe-OH b	38	81	71

H-Tyr-(*S*)-Ada-OH c	72	100	92
H-Tyr-(*R*)-Ada-OH c	88	99	97
H-Tyr-(*S*)-Ada-Gly-OH c	85	100	90
H-Tyr-(*R*)-Ada-Gly-OH c	87	100	89
H-Tyr-Ada-Gly-Phe-Met-OH c	85	100	85

a PG = percentage of growth at *c* = 10^−4^ M;

b data from Reference [12];

c data from Reference [11].

## References

[1] Otvos L. (2008). Peptide-based drug design: Here and now. Methods Mol. Biol..

[2] Purcell A.W., McCluskey J., Rossjohn J. (2007). More than one reason to rethink the use of peptides in vaccine design. Nature Rev. Drug. Discov.

[3] Lalive P.H., Neuhaus O., Benkhoucha M., Burger D., Hohlfeld R., Zamvil S.S., Weber M.S. (2011). Glatiramer acetate in the treatment of multiple sclerosis emerging concepts regarding its mechanism of action. CNS Drugs.

[4] Wilson A.C., Meethal S.V., Bowen R.L., Atwood C.S. (2007). Leuprolide acetate: A drug of diverse clinical applications. Expert Opin. Investig. Drugs.

[5] Wajcberg E., Tavaria A. (2009). Exenatide: Clinical aspects of the first incretin-mimetic for the treatment of type 2 diabetes mellitus. Expert Opin. Pharmacother.

[6] Zagon I.S., Verderame M.F., McLaughlin P.J. (2002). The biology of the opioid growth factor receptor (OGFr). Brain Res. Rev.

[7] Zagon I.S., Smith J.P., McLaughlin P.J. (1999). Human pancreatic cancer cell proliferation in tissue culture is tonically inhibited by opioid growth factor. Int. J. Oncol.

[8] Zagon I.S., Donahue R.N., Rogosnitzky M., McLaughlin P.J. (2008). Imiquimod upregulates the opioid growth factor receptor to inhibit cell proliferation independent of immune function. Exp. Biol. Med. (Maywood).

[9] Smith J.P., Bingaman S.I., Mauger D.T., Harvey H.H., Demers L.M., Zagon I.S. (2010). Opioid growth factor improves clinical benefit and survival in patients with advanced pancreatic cancer. Open Access J. Clin. Trials.

[10] Zagon I.S., Jaglowski J.R., Verderame M.F., Smith J.P., Leure-Dupree A.E., McLaughlin P.J. (2005). Combination chemotherapy with gemcitabine and biotherapy with opioid growth factor (OGF) enhances the growth inhibition of pancreatic adenocarcinoma. Cancer Chemother. Pharmacol.

[11] Horvat Š., Mlinarić-Majerski K., Glavaš-Obrovac Lj., Jakas A., Veljković J., Marczi S., Kragol G., Roščić M., Matković M., Milostić-Srb A. (2009). Tumor-cell targeted methionine-enkephalin analogues containing unnatural amino acids: Design, synthesis and *in vitro* antitumor activity. J. Med. Chem.

[12] Gredičak M., Supek F., Kralj M., Majer Z., Hollosi M., Šmuc T., Mlinarić-Majerski K., Horvat Š. (2010). Computational structure-activity study directs synthesis of novel antitumor enkephalin analogs. Amino Acids.

[13] Cherkasov A. (2005). Inductive QSAR descriptors. Distinguishing compounds with antibacterial activity by artificial neural networks. Int. J. Mol. Sci.

[14] Fjell D.C., Jenssen H., Cheung W.A., Hancock R.E.W., Cherkasov A. (2011). Optimization of antibacterial peptides by genetic algorithms and chemoinformatics. Chem. Biol. Drug Des.

[15] Cherkasov A., Janković B. (2004). Application of “inductive” QSAR descriptors for quantification of antibacterial activity of cationic polypeptides. Molecules.

[16] Hastie T., Tibshirani R., Friedman J (2009). High-dimensional problems: *p* >> *N*. The Elements of Statistical Learning: Data Mining, Inference and Prediction.

[17] Braga-Neto U., Dougherty E. (2004). Is cross-validation valid for small sample microarray classification?. Bioinformatics.

[18] Zhou X., Mao K.Z. (2006). The ties problem resulting from counting-based error estimators and its impact on gene selection algorithms. Bioinformatics.

[19] Chao S.M., Dougherty E.R. (2008). The peaking phenomenon in the presence of feature-selection. Pat. Rec. Let.

[20] Hua J., Xiong Z.H., Lowey J., Suh E., Dougherty E.R. (2005). Optimal number of features as a function of sample size for various classification rules. Bioinformatics.

[21] Tibshirani R. (1996). Regression shrinkage and selection via the lasso. J. Royal. Statist. Soc. B.

[22] Elad M (2010). Sparse and Redundant Representations—From Theory to Applications in Signal and Image Processingl.

[23] Tropp J.A., Wright S.J. (2010). Computational methods for sparse solution of linear inverse problems. Proc. IEEE.

[24] Kim S.J., Koh K., Lustig M., Boyd S., Gorinevsky S. (2007). An interior-point method for large-scale ℓ_1_ -regularized least squares. IEEE J. Sel. Top. Signal Proc.

[25] Ein-Dor L., Zuk O., Domany E. (2006). Thousands of samples are needed to generate a robust gene list for predicting outcome in cancer. Proc. Natl. Acad. Sci. USA.

[26] Yang F., Mao K.Z. (2011). Robust feature selection for microarray data based on multicriterion fusion. IEEE ACM Trans. Comp. Biol. Bioinforma.

[27] Muselli M., Bertoni A., Frasca M., Beghini A., Ruffino F., Valentini G. (2011). A mathematical model for the validation of gene selection methods. IEEE ACM Trans. Comp. Biol. Bioinforma.

[28] Kavuk O.D., Kamada M., Akutsu T., Knapp E.W. (2011). Prediction using step-wise L_1_, L_2_ regularization and feature selection for small data sets with large number of features. BMC Bioinforma.

[29] A repository of structures, experimental data and QSAR models for molecules with antitumor activity, 2008. Anticancer.irb.hr Repository Web site.

[30] Tetko I.V., Gasteiger J., Todeschini R., Mauri A., Livingstone D., Ertl P., Palyulin V.A., Radchenko E.V., Zefirov N.S., Makarenko A.S. (2005). Virtual computational chemistry laboratory—Design and description. J. Comput. Aid. Mol. Des.

[31] Sadowski J., Gasteiger J., Klebe G. (1994). Comparison of automatic three-dimensional model builders using 639 X-ray structures. J. Chem. Inf. Comput. Sci.

[32] Marvin—Draw and visualize chemistry, 2008. ChemAxon Web site.

[33] (2001). *l1_ls: Simple Matlab Solver for l1-Regularized Least Square Problem*, Beta Version.

