# Supplementary material for: Predicting Antitumor Activity of Peptides by Consensus of Regression Models Trained on a Small Data Sample
Source: Int J Mol Sci. 2011 Nov 29;12(12):8415–30. doi: 10.3390/ijms12128415 (PMC3257078; doi:10.3390/ijms12128415)
Supplement: Supplementary file 1 [file ijms-12-08415-s001.docx]

Supplementary Information

Predicting Antitumor Activity of Peptides by Consensus of Regression Models Trained on a Small Data Sample

Andreja Radman ^1^, Matija Gredičak ^1^, Ivica Kopriva ^2^ and Ivanka Jerić ^1,^*

^1^ Division of Organic Chemistry and Biochemistry, Ruđer Bošković Institute, Bijenička cesta 54, Zagreb HR-10000, Croatia; E-Mails: radman.andreja@gmail.com (A.R.);
matija.gredicak@irb.hr (M.G.)

^2^ Division of Laser and Atomic Research and Development, Ruđer Bošković Institute,
Bijenička cesta 54, Zagreb HR-10000, Croatia; E-Mail: ivica.kopriva@irb.hr

***** Author to whom correspondence should be addressed; E-Mail: ijeric@irb.hr;
Tel.: +385-1-4560-980; Fax: +385-1-4680-195.

Received: 11 November 2011; in revised form: 15 November 2011 / Accepted: 17 November 2011 /
Published: 29 November 2011

**Abstract:** Predicting antitumor activity of compounds using regression models trained on a small number of compounds with measured biological activity is an ill-posed inverse problem. Yet, it occurs very often within the academic community. To counteract, up to some extent, overfitting problems caused by a small training data, we propose to use consensus of six regression models for prediction of biological activity of virtual library of compounds. The QSAR descriptors of 22 compounds related to the opioid growth factor (OGF, Tyr-Gly-Gly-Phe-Met) with known antitumor activity were used to train regression models: the feed-forward artificial neural network, the *k*-nearest neighbor, the naïve Bayes, sparseness constrained linear regression, the linear and nonlinear (with polynomial and Gaussian kernel) support vector machine. Regression models were applied on a virtual library of 429 compounds that resulted in seven lists with candidate compounds ranked by predicted antitumor activity. The highly ranked candidate compounds were synthesized, characterized and tested for an antiproliferative activity. Some of prepared peptides showed more pronounced activity compared with the native OGF; however, they were less active than highly ranked compounds selected previously by the radial basis function support vector machine (RBF SVM) regression model. These results point to high complexity of prediction based on the regression models trained on a small data sample. The
ill-posedness of related inverse problem causes unstable behavior of trained regression models on test data.

**Keywords:** opioid growth factor (OGF), QSAR descriptors, consensus of predictors

**Table S1.** Cross-validation (CV) analysis over 10 × 100 random partitions for leave-one-out and leave-two-out. Reported are means and standard deviations of the correlation coefficient between true and predicted values. For each regression model correlation coefficient has been estimated on 10 blocks composed of 100 randomly chosen and predicted values. Means and standard deviations reported in the Table S1 are estimated from 10 correlation values representing corresponding blocks. SVM refers to support vector machine, RBF refers to radial basis function, KNN refers to k-nearest neighbors, and MLP ANN refers to multilayer perceptron artificial neural network.

| **Regression model** | **leave-one-out CV** | **leave-two-out CV** |
| --- | --- | --- |
| Sparse regression | 0.725 ± 0.035 | 0.716 ± 0.024 |
| Linear SVM, C = 2.6 × 10^3^ | 0.750 ± 0.026 | 0.529 ± 0.151 |
| RBF SVM, σ^2^ = 10, C = 10^4^ | 0.543 ± 0.058 | 0.481 ± 0.093 |
| Poly SVM, d = 2, b = 1, C = 250 | 0.549 ± 0.050 | 0.493 ± 0.100 |
| KNN, N = 1 | 0.453 ± 0.036 | 0.430 ± 0.094 |
| MLP ANN | 0.736 ± 0.036 | 0.696 ± 0.024 |

Table S2. (A) Correlations between features (molecular descriptors) and models’ predictions. Details about molecular descriptors are available at http://www.vcclab.org/lab/
indexhlp/. The right column reports on molecular descriptors that are maximally correlated with the prediction of corresponding regression model within a correlation interval specified for each regression model separately in the left column. The reason why correlation intervals are specified for each regression model separately is because maximal correlation levels between features and predictions vary from one regression model to another; (B) Correlations between features (molecular descriptors) and models’ predictions. The right column reports on top ten molecular descriptors that are maximally correlated with the prediction of corresponding regression model within a correlation level given in the parenthesis. SVM refers to support vector machine, RBF refers to radial basis function, KNN refers to k-nearest neighbors, and MLP ANN refers to multilayer perceptron artificial neural network.

(A)

| **Regression model** | **Molecular descriptor** |
| --- | --- |
| Sparse regression: 0.7 < corr < 0.75 | piID, MATS4p, Mor20m |
| Linear SVM: 0.7 < corr < 0.75 | Mor20m |
| MLP ANN: 0.65< corr < 0.7 | Mor20m, HATS2v, HATS3v |
| RBF SVM: 0.7 < corr < 0.75 | n404, n408, SRW04, SRW06, 2RW08, HOMA, ntH,  C-032, C-033, H-048, N-071 |
| Poly SVM: 0.7 < corr < 0.8 | nBO, nC, ZM1, ZM2, GMTI, W, w, D/Dr12, T(N..N), piPC01, piPC02, piPC03, piPC04, piPC07, piPC10, TPC, PCR, QZZp, RDF015e, Mor06m, C-024, P-118 PX3 |
| Poly SVM: 0.75 < corr < 0.8 | TIC2, D/Dr12, TPC, QZZp, Mor06m, P-118 PX3 |
| Poly SVM: 0.8 < corr < 0.85 | TPC (0.833) |
| KNN | no feature with correlation greater than 0.4 |

Table S2. *Cont*.

(B)

| **Regression model** | **Molecular descriptor** |
| --- | --- |
| Sparse regression | MATS4p(0.728), Mor20m(0.722), piID(0.713), Km(0.655), Mor15v(0.649), PCR(0.645), HATS2v(0.630), HOMA(0.629), MATS5p(0.626), Mor10e(0.621) |
| Linear SVM | Mor20m(0.722), HATS3v(0.674), MATS5p(0.667), HOMA(0.664), piID(0.663), SRW06(0.656), nR04(0.656), nR08(0.656), SRW04(0.656), SRW08(0.656) |
| MLP ANN | Mor20m(0.684), HATS3v(0.683), HATS2v(0.669), HATS3p(0.632), HATS2p(0.628), HATS2m(0.624), HOMA(0.618), SRW06(0.618), nR04(0.618), nR08(0.618) |
| RBF SVM | HOMA(0.714), SRW06(0.706), SRW08(0.706), nR04(0.706), nR08(0.706), SRW04(0.706), ntH-Thiophenes(0.706), C-032(0.706), C-033(0.706),  H-046(0.706) |
| Poly SVM | TPC(0.833), Mor06m(0.774), nC(0.773), D/Dr12(0.764), QZZp(0.752),  P-118(0.752), piPC04(0.750), ZM1(0.743), ZM2(0.742), piPC02(0.737) |
| KNN | Mor20m(0.394), HATS2v(0.362), HATS3v(0.360), EEig01x(0.347), HATS2p(0.345), HATS3p(0.340), Mor15p(0.340), HATS2u(0.336), R6u+(0.336), Mor15v(0.335) |

Figure S1. MS/MS spectrum of the molecular [M + H]^+^ ion of peptide 10.


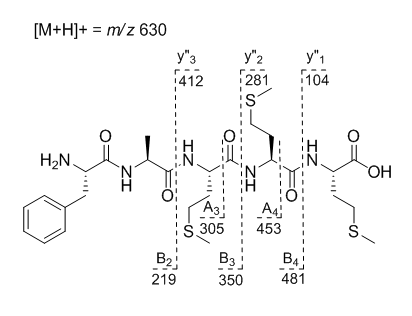

10

Figure S1. *Cont.*


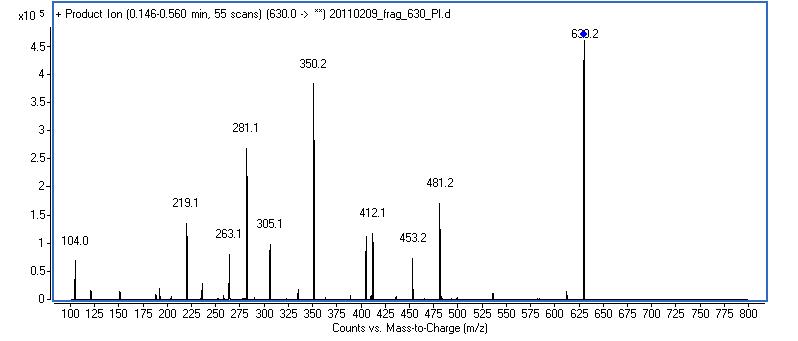


© 2011 by the authors; licensee MDPI, Basel, Switzerland. This article is an open access article distributed under the terms and conditions of the Creative Commons Attribution license (http://creativecommons.org/licenses/by/3.0/).
